# A Systematic Review on the Generation of Organic Structures through Additive Manufacturing Techniques

**DOI:** 10.3390/polym16142027

**Published:** 2024-07-16

**Authors:** Alex Bernadi-Forteza, Michael Mallon, Christian Velasco-Gallego, Nieves Cubo-Mateo

**Affiliations:** 1 Research Group ARIES, Higher Polytechnic School, Nebrija University, 28040 Madrid, Spain; cvelasco@nebrija.es (C.V.-G.); ncubo@nebrija.es (N.C.-M.); 2 European Space Research and Technology Centre, European Space Agency, 2201 AZ Noordwijk, The Netherlands; michael.mallon@esa.int

**Keywords:** stochastic structures, additive manufacturing, bone printing, supportless, self-generated

## Abstract

Additive manufacturing (AM) has emerged as a transformative technology in the fabrication of intricate structures, offering unparalleled adaptability in crafting complex geometries. Particularly noteworthy is its burgeoning significance within the realm of medical prosthetics, owing to its capacity to seamlessly replicate anatomical forms utilizing biocompatible materials. Notably, the fabrication of porous architectures stands as a cornerstone in orthopaedic prosthetic development and bone tissue engineering. Porous constructs crafted via AM exhibit meticulously adjustable pore dimensions, shapes, and porosity levels, thus rendering AM indispensable in their production. This systematic review ventures to furnish a comprehensive examination of extant research endeavours centred on the generation of porous scaffolds through additive manufacturing modalities. Its primary aim is to delineate variances among distinct techniques, materials, and structural typologies employed, with the overarching objective of scrutinizing the cutting-edge methodologies in engineering self-supported stochastic printable porous frameworks via AM, specifically for bone scaffold fabrication. Findings show that most of the structures analysed correspond to lattice structures. However, there is a strong tendency to use organic structures generated by mathematical models and printed using powder bed fusion techniques. However, no work has been found that proposes a self-supporting design for organic structures.

## 1. Introduction

Bone grafts serve as vital substitutes for damaged or lost tissue, providing cellular support and material replenishment [1]. Extensive research has delved into how the scaffold porosity, in terms of pore volume, density and morphology, affects bone regeneration [2,3,4,5,6]. Crucially, scaffold osteogenic potential hinges upon inter-pore connectivity, facilitating cellular dispersion, tissue integration, and vascular ingrowth. Therefore, the preparation of bone scaffolds with applicable pore size and interconnectivity is an important issue in bone tissue engineering. However, to be effective in vivo, the scaffold must also meet physiological mechanical loading requirements [7]. 

Regarding bone porosity, optimal tissue growth and scaffold functionality necessitate pores with an average diameter of 100 μm, because osteoclasts have that dimension and must be able to pass through the pores, and it also necessitates porosity levels between 50 to 90%, mirroring trabecular bone characteristics [8]. On the other hand, when pores are of small sizes, it is possible to better control cell aggregation and proliferation [9]. Abbasi et al. reported that a pore size in the range from 200 to 350 µm was ideal for osteoblast proliferation, while a larger pore size (500 µm) did not affect cell attachment [7]. This review also mentioned that other studies show that a pore size of 800 µm was more appropriate to provide adequate space for cell growth. 

From the above-mentioned literature, it could be extrapolated that currently designed scaffolds with small pore sizes (i.e., <200 μm) show in vitro and in vivo osteoblast survival and bone formation limited to the periphery, due to less diffusion of oxygen and nutrients into the scaffolds. At the same time, scaffolds with a mean pore size of 300 μm show increased proliferation and differentiation of osteoblasts throughout the scaffold, due to increased neovascularization and massive oxygen and nutrient transport. Big pore sizes (>300 μm) can ensure effective scaffold function for bone regeneration. However, although high porosity and pore size may be favourable, it should be considered that high values condition the load-bearing requirement. Also, as summarized in Table 1, each bone cell has a different size and requires specific dimensions to carry out its function. 

Consequently, a combination of micro- and macropores is imperative to meet both structural and biological requisites for bone regeneration. Traditional tissue engineering methods have limitations in generating such structures, precluding the deposition of cells throughout the scaffold fabrication process [11]. Hence, emerging technologies like additive manufacturing (AM), or 3D printing, have garnered attention for their ability to create customized porous structures. However, prevalent AM techniques often yield regular lattice structures, which may compromise scaffold performance due to their susceptibility to crack propagation.

AM has been featured as a trendy technology in this field, due to its capacity to create tailored porous structures [1]. However, most of the additive manufacturing slicing software finally presents a low variety of infill patterns [12]. That is, most commonly replicated structures found in the literature are regular, repetitive cell units. These repeating prismatic structures are easier to produce and deposit through micro-extrusion with thermoplastics and even hydrogels and pasty materials used in bioprinting [13]. Although the distance between the deposited strands and other parameters characteristic of each pattern can be modulated, crossed or combined, the final result is still a regular lattice.

Regular lattices can bias the direction of cracking because they frequently offer low energy paths for element breakdown, provoking a deep fracture in the materials [14]. These structures present poor behaviour against crack propagation, due to their regularity, and propagate through the planes in a fast and deep way. This collapse is unacceptable, mainly in scaffolds that will be introduced in vivo before complete maturation or regeneration. 

For this reason, researchers are actively addressing these limitations by exploring novel infill patterns and advancements in AM software and hardware. The forecast is toward more complex and optimized structures that balance mechanical properties and porosity, obtaining better performance in load distribution and crack propagation. 

In contrast, some studies have shown that stochastic or organic structures can exhibit self-limiting crack propagation, meaning that the crack growth rate decreases or stops as the crack length increases. This can be due to the presence of multiple paths for the crack to follow, the variation in the local stress intensity factor, or the interaction of the crack with the microstructure. 

Bone structure is a complex and hierarchical material that has evolved to resist crack propagation and enhance fracture toughness. Its structure has various features that can deflect, bridge or stop cracks at different scales. At the microscale, this is achieved through the osteons, which are cylindrical units of concentric lamellae of mineralized collagen. The osteons are surrounded by cement lines, which are thin layers of nonmineralized, or poorly mineralized, collagen. The cement lines act as weak interfaces that can deflect or arrest cracks that propagate from the interstitial bone. Osteons also have different orientations and arrangements, which create a heterogeneous and anisotropic structure that can resist cracks from different directions. 

Conversely, stochastic or organic structures have shown promise in mitigating crack propagation through self-limiting mechanisms. Mimicking the hierarchical features of bone structure, such as irregularities, gradients and reinforcements, can enhance scaffold resilience. Nonetheless, creating such organic structures poses challenges in modelling, optimization and quality control. 

This systematic review undertakes a comprehensive examination of the current landscape of 3D bone printing technology. It aims to dissect the methodologies utilized in generating these structures, encompassing both structural typology and printing methodology. Emphasis is placed on identifying research endeavours focused on crafting organic structures conducive to 3D printing without supplementary support structures. The ultimate objective is to ascertain whether existing research has proposed algorithms capable of generating self-supported stochastic porous structures suitable for 3D printing.

## 2. Methods

This systematic review was conducted following the Preferred Reporting Items for Systematic Reviews and Meta-Analyses (PRISMA) guidelines [15]. The process used to carry out this systematic review consists of the following five steps:

- **Phase 1: Identification of the research questions**: Determine which will be the questions to be answered for this systematic review. The questions should be able to gather the main ideas of this study, arriving at a synthesis of the current state of the art about this review seeks to satisfy.
- **Phase 2: Definition of the search strategy**: Find the keywords that best represent the main ideas raised in the research questions. In addition, this phase determines how to use logical operators to achieve a more precise search.
- **Phase 3: Definition of the inclusion and exclusion criteria**: Establish the limits of the search by defining the conditions that must be met by all items to be considered in the review.
- **Phase 4: Screening of the primary studies**: Eliminate from the study the articles that do not meet the criteria defined in the previous phase.
- **Phase 5: Data extraction**: Define the data to be extracted from the resulting articles, synthesized in the Results section, and used to answer the research questions.

### 2.1. Identification of the Research Question

The research questions of this study have been defined to collect the current methods used for the generation of porous structures with additive manufacturing. Special attention has been paid to the generation of non-regular porous structures fabricated by Fused Deposition Modelling (FDM) and the possible existence of self-generated designs. Although the goal of the study is focused on 3D bone printing, in other to reduce the limitations of this review, the possibility that there may be methods outside of this field that allow the generation of porous structures, which can be extrapolated to the generation of bone scaffolds, is not to be excluded. For this reason, the focus of the review was broadened to include articles that may not be related to bone printin, but that propose porous structures. According to this, the following research questions are addressed:

- **Research Question 1 (RQ1):** “What are the main types of mesoscopic structures for the generation of porous structures?”
- **Research Question 2 (RQ2):** “What are the most common methods used to generate stochastic porous structures?”
- **Research Question 3 (RQ3)—Regarding Research Question 2:** “What are the 3D printing methods used to generate non-regular structures?”
- **Research Question 4 (RQ4):** “What proposals exist for the generation of self-generating stochastic structures?”

The infill of a porous structure can be classified as regular or non-regular. A structure is considered to be regular, or lattice if it has been obtained by the periodic repetition of a unit cell. On the contrary, if there is no type of pattern in the internal structure, the structure is non-regular or stochastic. That is why the RQ1 aims to identify what type of infill is commonly used to generate porous structures and what the types of unit cells used for it are. RQ2 aims to investigate the state of the art of the design of stochastic structures whether using an algorithm, Computer-Aided Design (CAD) or any kind of novel method. RQ3 tries to analyse if the proposed stochastic structures can be printed in 3D. The objective of this question is to know if any proposed methods can generate stochastic structures that can be used as bone scaffolds. For this, it is necessary to know if the proposed design can be 3D printed using a method that is compatible with the use of living bioinks. Finally, the last question (RQ4) aims to investigate the generation of self-generated structures. A self-generated structure is defined as a structure that, given boundary or structural conditions and a volume, generates itself according to these conditions.

### 2.2. Definition of the Search Strategy

This phase aims to identify the keywords and the research resources to be used in the research. Initially, a couple of simple searches were performed to find out what the best keywords to use would be by seeing which keywords returned articles related to our research and seeing which keywords were used in these related articles. The keyword combinations used for the initial searches and their results are shown in Table 2.

This preliminary search allowed the most representative keywords to be deduced. In addition, a synthesis of the different keywords of the articles obtained in the preliminary search was carried out to have a general idea of the keywords used in the articles of interest. They were added in a more complex search to find as many studies as possible that are related to the topic of this review.

All the featured keywords extracted from these initial searches can be classified in a total of three distinguished main ideas, summarized in Table 3. 

It is acknowledged that there are studies potentially related to the objective of this research that cannot be taken into account due to the use of synonyms of the proposed keywords or other terminology, which may represent a case of bias. However, it is believed that the keywords used to represent the main ideas of the research are the most appropriate, as they were obtained from initial keyword searches. In addition, since the keywords are extracted from the articles, the bias that the authors might contribute is reduced.

The research was performed using the SCOPUS database. A complex search was performed where each main idea was represented by all extracted keywords related to it linked by the Boolean operator OR, and, in turn, each main idea is connected by the Boolean operator AND. Wildcards of some of these keywords were used to avoid not including articles due to an unusual way of writing the keywords. Also, loose phrases were used in almost all keywords to cover as many documents as possible. In addition, the search was limited to a time range between 2013 and April 2024. The query string used was the following:

“(TITLE-ABS-KEY (“bone*”) OR TITLE-ABS-KEY (“porous material”) OR TITLE-ABS-KEY (foam*) OR TITLE-ABS-KEY (open-cell) OR TITLE-ABS-KEY (“porous structure*”) OR TITLE-ABS-KEY (“complex structure*”)) AND (TITLE-ABS-KEY (“additive manufacturing”) OR TITLE-ABS-KEY (bioprint*) OR TITLE-ABS-KEY (“3d printing”) OR TITLE-ABS-KEY (fdm) OR TITLE-ABS-KEY (“geometric modeling”)) AND (TITLE-ABS-KEY (“BONE-LIKE”) OR TITLE-ABS-KEY (“no* lattice”) OR TITLE-ABS-KEY (“organic structure”) OR TITLE-ABS-KEY (“stochastic”) OR TITLE-ABS-KEY (“no* regular”) OR TITLE-ABS-KEY (“modeling approach”) OR TITLE-ABS-KEY (“no* parametric design”) OR TITLE-ABS-KEY (“network-based”) OR TITLE-ABS-KEY (“graph-based”) OR TITLE-ABS-KEY (“inhomogeneous porous structure*”) OR TITLE-ABS-KEY (“irregular internal morphology”)) AND PUBYEAR > 2013”.

### 2.3. Definition of the Inclusion and Exclusion Criteria

To ensure that all articles to be analysed were within the scope of this review, exclusion criteria were included (Table 4). This criterion enables the systematic exclusion of studies that, even if they are related to the topic of this review, may not be of interest. 

### 2.4. Screening of the Primary Studies

The results obtained are analysed by title and abstract to discriminate between those that meet the inclusion criteria and those that do not.

**Figure 1:**
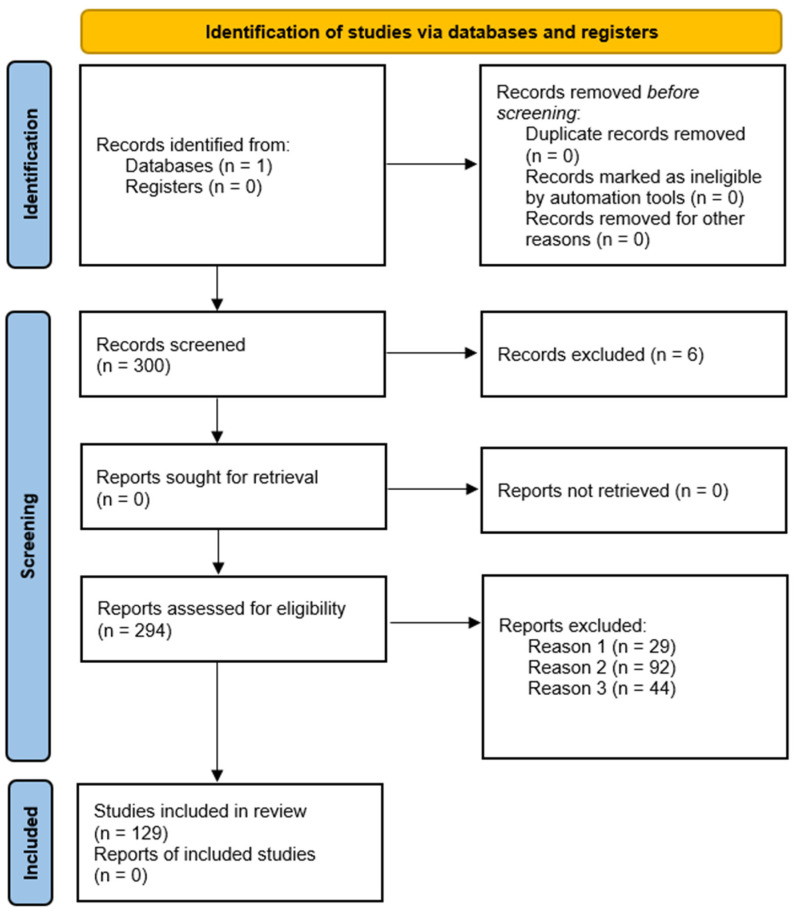
PRISMA flowchart showing the steps of the article selection process with numbers of included and excluded articles.

There were 300 documents from this keyword search. Among them, six were written in Chinese, which were discarded from the study. Failure to consider articles written in languages other than English could be considered a case of bias, but the authors are not familiar with those languages. These 294 articles were screened to discern which papers were eligible for inclusion in the study by two people to reduce potential bias. Articles excluded by a single person were analysed by an independent third person (Table 4). A total of 165 articles were excluded according to these criteria, resulting in 129 articles included in the review (Figure 1).

### 2.5. Data Extraction

Each of the 129 articles were analysed, and the data necessary to answer each of the research questions were extracted. In addition, other data were extracted that did not address the questions but were considered interesting to characterize each of the articles. The data extracted are summarized in Table 5 together with the research question to which they refer.

## 3. Results

In this section, the data extracted from the articles are summarized and analysed to answer the research questions. The data are grouped as they were in the previous section.

### 3.1. Printing Method

These one hundred and twenty-nine articles propose diverse types of porous structures for a variety of purposes. Of these one hundred and twenty-nine, 83.7% (n = 108/129) print the structures they propose. While 16.3% (n = 21/129) only model the structures in 3D using CAD. The methods used to manufacture the structures in the articles are very varied. They are grouped according to the physical process that characterizes them in Table 6. Some advantages and disadvantages of each for the manufacture of porous structures are shown in Table 7.

The most common printing processes are powder bed fusion (41.1% n = 44/107) followed by material extrusion (34.6% n = 37/107). This contrasts with the most used printing method being FDM (15% n = 16/107), corresponding to material extrusion, followed by SLS (11.2% n = 12/107). This contrast reflects the number of different printing methods that exist within the powder bed fusion process.

Among the 129, there are 47 articles related to bone bioprinting. Among them, the most used printing methods are material extrusion and powder bed fusion. Both are used the same number of times (n = 18/47). Material extrusion is widely used because the materials with the best biocompatibility for osteoblast proliferation, such as biphasic calcium phosphate (BCP) or polycaprolactone (PCL), are polymeric materials [17]. Powder bed fusion is highly used because all articles proposing medical prosthesis designs usually use titanium, or titanium alloys, as the base material. Titanium and titanium alloys are used to manufacture scaffolds because they present good biocompatibility, high strength-to-weight ratio, corrosion resistance, durability and strong osteointegration for the proliferation of osteoblasts [83,108,109,117]. Its integration, as a transplant of a bone section has some disadvantages such as the generation of shear stress due to the difference in elastic modulus between titanium and bone [110]. These shear stresses cause a decrease in the osseous material and can lead to osteoporosis [38,113,118,123]. The main aspects of each method concerning the biocompatibility of these methods are summarised in Table 8.

Regarding the articles that propose no-lattice structures, most of the proposed no-lattice structures are printed (82.2% n = 37/45), with PBF (56.8% n = 21/37) being the most commonly used printing method. Again, titanium, alloyed or not, is the most used material (66.7% n = 14/21), although the use of ABS (SL resin) [82], PCL [72] and nylon [99] in some articles is surprising for its uncommon use in the PBF process. 

### 3.2. Structure

To classify the type of structure that a porous material presents, the geometry of its unit cell and the periodicity of the unit cells were analysed. If it presents an internal periodicity, the material is considered lattice; otherwise, it is considered no-lattice or stochastic. For this classification, no distinction is made as to whether the structure is printed or not.

The 65.1% (n = 84/129) of the structures generated in the literature are lattice versus 34.9% (n = 45/129) that are stochastic. Lattice structures can be grouped into geometrical (the unit cell corresponds to a geometric figure), analytic (the unit cell is generated with a mathematical expression) and grid (the is not a unit cell, the structure is generated stacking material threads). 

The geometric unit cell is the most commonly used, 63.1% (n = 53/84), for designing the lattice structures. The most used figure is cubic followed by the octet, Kelvin and hexagonal. But there are also some articles [87,107,113] that use structures based on the cubic crystal system and have been included in the geometric group as they are modifications of a cube. Seetoh et al. [32] proposed an auxetic 3D anti-tetrarchical (3ATC) figure, a member of the joint-rotation-dominated cellular topology, as a unit cell. This is a member of the joint-rotation-dominated cellular topology and was classified inside the geometric group because it mainly consists of six struts connected to a central cubic joint. Additionally, other authors propose using fractals to mimic cortical bone structure. Periodic fractal rings inspired by the Koch snowflake fractal are stacked to generate a radial-gradient scaffold [48]. It has been considered to belong to this group because its structure is achieved by performing transformations to initial geometric structures, although in this study there is no unit cell per se. All the structures in this group are generated using different CAD softwares.

The analytic group is completely dominated by the triply periodic minimal surface (TPMS) unit cells (15.5% n = 13/84). TPMS structures are generally used due to their flexible lattice structure design, well-defined analytical expression, anisotropic features [27], high specific surface area, excellent additive manufacturing properties [86], high stiffness-to-weight ratio and connected pores [61].

**Figure 2:**
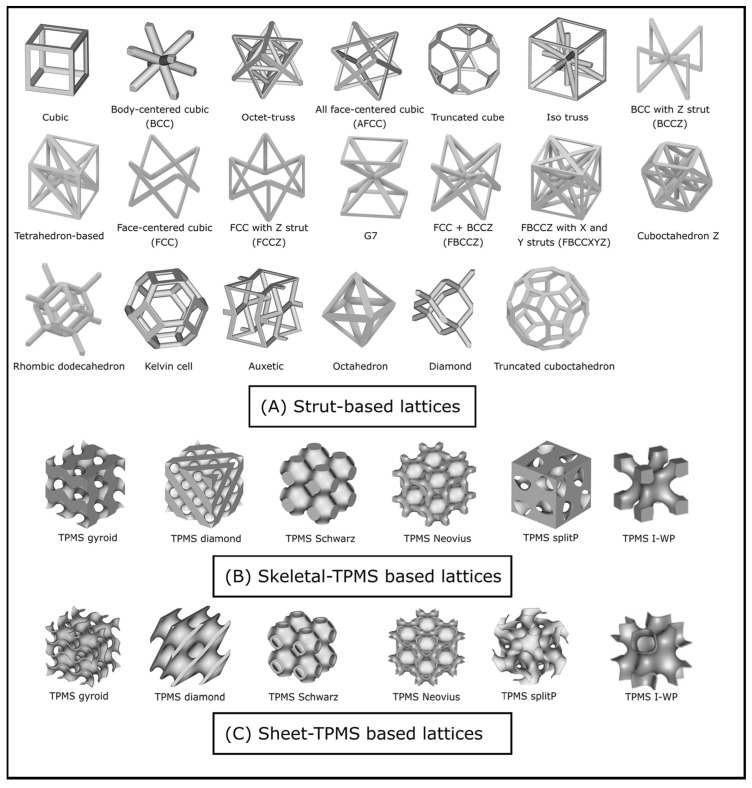
Various lattice unit cells (**A**) Strut-based or geometrical lattice (**B**,**C**) TPMS-based unit cells. Image extracted with Creative Commons CC-BY-NC-ND 4.0 permissions from Plessis et al. Figure 2 [124].

Grid structures are mainly used to manufacture bone scaffolds. Of the 19 articles using the grid structure, 13 of them are related to the construction of bone scaffolds. This is because they are mainly articles that focus more on the development of materials with high biocompatibility than on the structure they generate [44,46,47,53,62,69,70,72,74,76,100,104]. An example of a variety of the regular unit cells used is shown in Figure 2.

Stochastic structures are proposed in just 34.9% (n = 45/129) of the articles. As stochastic structures do not have a unit cell, they have been classified according to the approach used to be created. Several design approaches were proposed to create stochastic cellular materials. For example, Simoneau et al. [114] propose a multiscale approach that consists of dividing the structure into small cubes and removing a specific number of them at random. Patterson et al. [16] create a lattice structure with tetrahedrons and then remove random struts to study the effect of missing struts on the mechanical properties of the structure. Wang et al. [83] created random structures deforming a lattice unit cell that they propose. Ullah et al. [121] propose an IFS-based algorithm to create fractal geometry-based realistic porous structures. Other articles generate non-regular porous structures by dividing a volume into, generally, Voronoi subspaces that they fill with different types of TPMS [82,99,102,105,125]. These studies have been grouped in a group called modified lattice.

**Figure 3:**
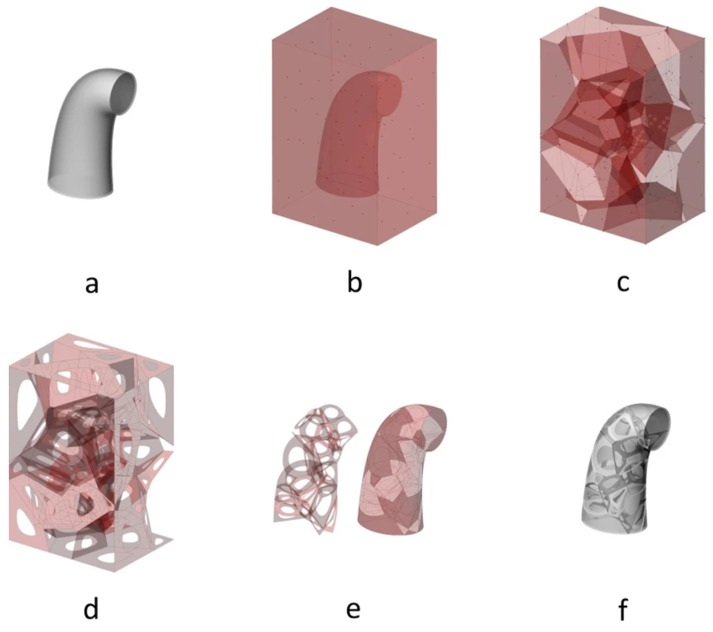
From **a** to **f**, the process of designing a porous structure within a desired volume from the intersection of that volume and the cells of a Voronoi diagram generated in a larger volume. Image reused with Creative Commons 4.0 CC BY permissions. Example of workflow followed to generate the porous structure found in Figure 6 of Piros et al. [126].

**Figure 4:**
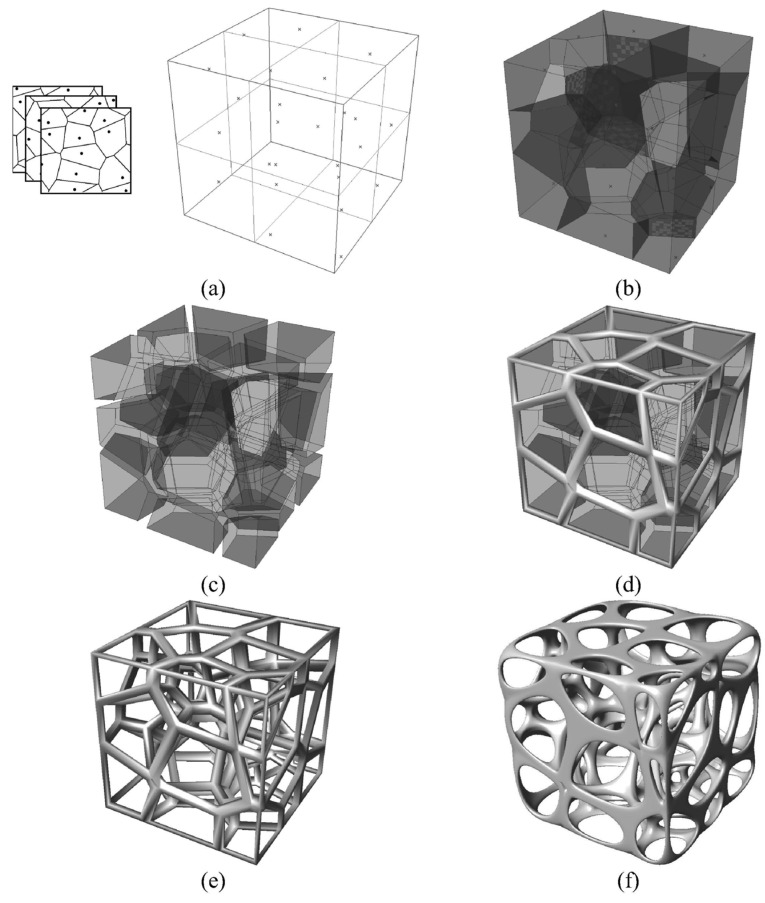
Process to obtain 3D Voronoi cell regular porous structures. (**a**) Two-dimensional Voronoi point coordinates (x,y) are processed at equal z distances to obtain the 3D coordinates (x,y,z) of all points. (**b**) Then, the coordinates are processed to obtain the 3D Voronoi cell structure. (**c**) Then, each plane surface is self-copy and translated a fixed distance to create an open internal connected volume. (**d**,**e**) Then, the porous external (**c**) and internal (**d**) contours are obtained by Boolean operations. (**f**) Finally, a smoothing process is performed to soften the trabecular mesh model. Reused with permissions. Example of Voronoi-based generation of stochastic structure by Gómez et al. Figure 3 [131].

Another alternative used to generate these stochastic structures is using Voronoi spaces. The general procedure used is to fill a design space with random points (called seeds), generate Voronoi volumes from these seeds and generate a structure from the intersection of the different Voronoi volumes. The difference between each study lies mainly in the way these seeds are generated. Piros et al. [126] imported the 3D model into a larger space, in which seed points are randomly placed and Voronoi cells are generated. From these, they calculate the intersection between the initial geometry and the Voronoi cells and thus obtain the filling of the initial structure, as shown in Figure 3. Other authors generate seed points randomly according to the desired final pore size [31,38,127,128,129]. Alsheghri et al. [130], Vlad et al. [101] and Gómez et al. [131] base the location of the seed points on information extracted from bone by micro-computed tomography (µCT). By way of example of one of these three methods, the approach proposed by Gómez et al. [131] is shown in Figure 4. The centre of the pores is extracted from µCT at different heights and then transferred to a 3D volume where they place the seed centres from which to grow the Voronoi cells.

**Figure 5:**
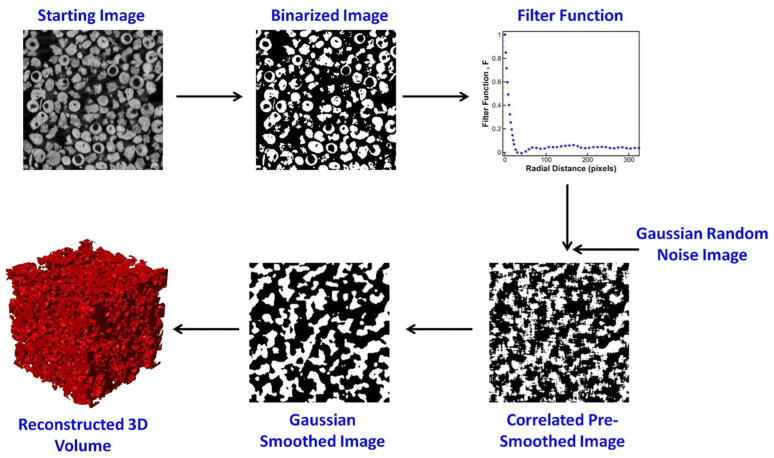
From top left to bottom left, workflow followed to reconstruct the 3D porous volume from glass beads µCT images. Reused with permissions. An example of μCT-based workflow to generate stochastic structures proposed by Karthik et al. Figure 2 [133].

On the other hand, other authors use µCT to generate porous structures from other structures. The methodology used is the same in all articles and consists of scanning a porous structure by µCT to obtain its 3D CAD model. What differentiates each approach is the type of structure that is scanned. Baino et al. [18] use the tomographic reconstruction of a polyurethane sponge. Baino et al. [19] obtained 3D porous scaffolds resembling the architecture of cancellous bone. Berger et al. [77] scanned a human femoral head retrieved from a hip replacement. Matheson et al. [93] used an aluminium open-cell foam produced by investment casting. Homberg et al. [132] adapted realistic trabecular structures. Hernández-Nava et al. [119] and Bodla et al. [133] made use of randomly packed beds of glass beads. Those glass beads are scanned by µCT, and a Gaussian filter is applied to the resulting images to obtain continuous structures. Finally, the 3D volume is reconstructed from the modified µCT images. This process can be seen in Figure 5.

The last and most common (32.4% n = 12/37) of the methods used in the literature to generate non-regular structures was called “stochastic”. It is characterized by the fact that the structures are completely stochastic and do not come from any previous figure or structure. Li et al. [96] and Ambekar et al. [49] used the Cahn–Hillard equation to model a spinodal decomposition to generate stochastic bicontinuous porous structures. Gu et al. [90] used the software 3-Matic (Materialise, Leuven, Belgium) to topologically optimize a cylinder and obtain a porous structure. Other authors filled a volume with points in a pseudo-random way to ensure a minimum distance between the points, which were then joined together under some conditions such as the maximum number of connections per node, the minimum length of the connections or the minimum angle of the connections [84,89,92,94]. Refs. [26,42,95] also filled the volume with random points, but instead of joining them, they used them as seeds to create spheres with the desired volume and remove them from the volume, creating a porous structure. Sharif Ullah et al. [122] created an annular porous structure from radial sections of a cylinder in which they distribute points using Monte Carlo and then applied concave hull operations to create a 3D structure. Zhang et al. [128] created an initial structure by generating random points inside a desired volume and joining them following a connection criterion. Then, the Coulomb repulsion and Hooke attraction forces were calculated for each node’s to update each node position until an equilibrium was reached and the optimization process converged. Four articles propose self-developed topological optimization algorithms based on principal stresses to obtain stiffness-optimized porous structures [54,65,71,134].

Regarding the stochastic structures that have been developed for the study of bone bioprinting, only 15 articles are related to bone printing, and the main methods used to generate the structures are Voronoi ([38,79,101,116,130]) and µCT ([18,19,77]). Kohli et al. and Kechagias et al. [84,85], generated a stochastic structure on which it is possible to influence the parameters that characterize it (length of the joints, number of joints, pore size, etc.). Voronoi is not a controllable method since the only parameter that is defined is the number of seeds. The methods proposed by [26,42,95] can also control the pore size in addition to the number of seeds. The other two papers are related to bioprinting and use regular structure deformation and topological optimization, respectively, to generate the structures [83,90].

### 3.3. Three-Dimensional Model Generation

The procedure of all articles can be summarized in three steps: (a) design a 3D model of the proposed structure, (b) manufacture the structure and (c) perform a quasi-static mechanic resistance test. Articles that do not manufacture the proposed structure follow a two-step procedure: (a) design a 3D model of the proposed structure and (b) Finite element methods (FEM) analysis to determine the mechanical properties. References [29,41,57,59,61,89,106,107,108,111,114,118,135] perform a different last step simulating and performing quasi-static tests of the structure to compare both results. Generally, this is carried out to verify if the mechanical behaviour of the structure can be predicted by some mathematical model proposed, like Gibson–Ashby [27,29,61,89,114,118] or Hashin and Shtrikman [33]. Ref. [115] went one step further and generated a data-driven model from different TMPS topologies and used it to train a generative adversarial network (GAN) to learn the mapping of material properties from geometric parameters.

Among all articles, 52.0% (n = 67/129) do not specify how the 3D model was created or the workflow followed to create it. Moreover, 58.1% (n = 75/129) do not specify which CAD software was used, and 37.2% (n = 48/129) of the articles do not indicate either the method used to generate the model or the software used. Only the 27.1% (n = 37/129) articles specify the algorithm used and the software used. Among them, 16 articles use a lattice structure. So only 19.0% (n = 16/84) of the articles that propose a lattice structure properly explain the methods used to generate the CAD model. Although this value is low, it is understandable since most regular structures are created with predefined figures, usually in CAD software. On the other hand, all the articles proposing stochastic structures explain the method used to generate them, but only 46.7% (n = 21/45) of them specify the software used. The software used to generate stochastic structures can be divided into calculation/programming or CAD environments. In the first group, Matlab, Wolfram Mathematica and Python are found. And the second group includes Rhinoceros 3D, Autodesk, SolidWorks, nTopology, 3-Matic and Creo Parametric. Both are ordered according to frequency of use.

The trend mentioned above for stochastic structures continues for all types of structures, as Matlab and SolidWorks are the most used (44.4% n = 24/54) software to generate the 3D model. Both used the same number of times. Followed by Rhinoceros 3D used in 16.7% (n = 9/54) articles.

### 3.4. Self-Supporting Structures

While it is true that regular structures are largely unsupported 3D-printable, as they are based on geometric patterns, which use edges that do not have critical angles and do not overhang [63,68], or on TPMSs, which have a surface that has a shallow curvature that itself serves as a support for printing future layers [82]. However, there may be designs that suffer deformations due to the lack of support material [82]. The vast majority of stochastic structures have edges with steep angles that are not 3D printable without support. 

The critical printing angle, beyond which the use of support material will be necessary, will depend on both the material and the printing method. The smaller the critical angle, the less restriction there will be when printing and the more complex the structures to be printed can be. Hossain et al. [92] printed titanium using PBF and were able to print unsupported up to an angle of 15°, while Carneiro et al. [63] printed polylactic acid (PLA) using FFF and were able to print angles up to 30°.

Self-supported printing is not a main design requirement of the analysed articles, as only nine articles mention it. There are only two articles that generate structures intended to be printable without support: one proposes a regular structure [118] and the other a stochastic structure [65]. 

Zhao et al. [65] propose an algorithm that includes two optimization processes: first, the part is topologically optimized to improve the mechanical properties, and then, a second optimization is applied to eliminate those parts that prevent the part from being printed without support. The second filter is dependent on the printing direction that the part will have, so it is necessary to know a priori how the printing will be carried out.

Wu et al. [54] propose a topological optimisation algorithm to generate the stochastic structure. The structure they propose as an example could be made printable without support, but as this is not an aspect they consider in the optimisation process it cannot be assured that any structure generated by the proposed algorithm can be made printable without support. Refs. [21,26,37,66,102,115,116] mention that the proposed structures are fabricated without support as they use printing methods where the part is immersed in fluid or powder, and it is this material that acts as a support. Even so, they do not consider the absence of support material as a design parameter for their structures, so they could only be printed using methods that involve immersing the part in a medium.

### 3.5. Overview

**Figure 6:**
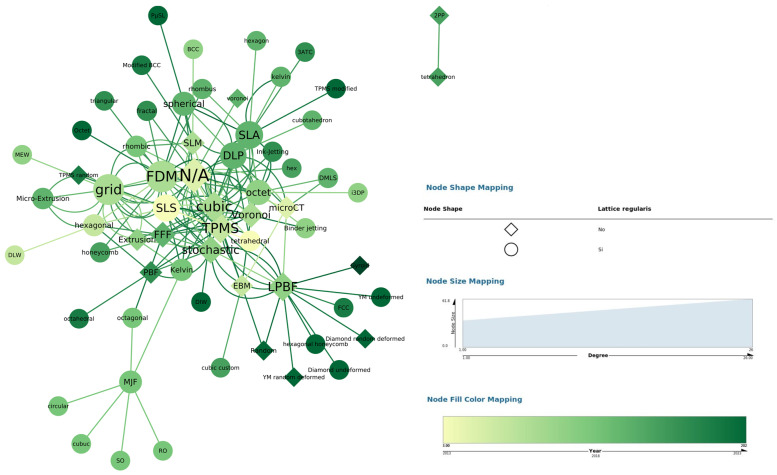
Time-relational graph of the different printing methods used and the types of structures used.

To have a global understanding of the main aspects extracted in this review, a graph has been generated that relates the following aspects: type of cell unit and printing method. The size of the nodes depends on the number of occurrences of the particular concept in the articles analysed, and the shape of the nodes depends on the type of structure used. In addition, an extra time variable has been added to study the variation in the trends used over time. Thus, four independent aspects can be seen related in Figure 6.

## 4. Discussion

In this section, the information extracted in the previous section is used to answer the research questions, and then the findings are exposed.

### 4.1. Answer to RQ1: What Are the Main Types of Mesoscopic Structures for the Generation of Porous Structures?

The main types of mesoscopic structures for generating porous structures are reticular and stochastic structures. There is a clear preference for the use of reticular structures, which are used in two out of three articles. Among all of them, the most used structure is the cubic lattice.

### 4.2. Answer to RQ2: What Are the Most Common Methods Used to Generate Stochastic Porous Structures?

**Figure 7:**
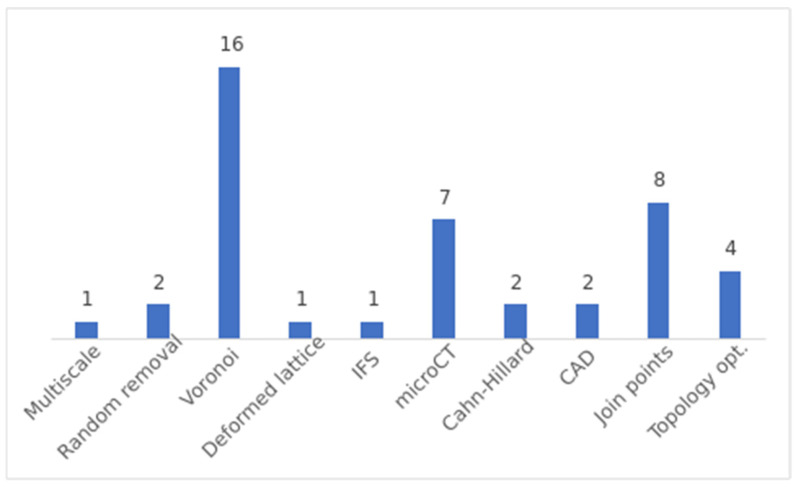
Frequency of use of the different methods to generate stochastic structures.

The different methods used to generate stochastic porous structures found in the literature and their frequency are shown in Figure 7.

Therefore, the most common methods in the literature are the following:

- Voronoi-based Approaches: Voronoi spaces are commonly used to generate stochastic structures. The general procedure involves filling a design space with random points or seeds and generating Voronoi spaces from these seeds. The resulting structure is formed by the intersection of the different Voronoi spaces. The specific method for generating the seeds can vary, including random placement in a larger space or using information extracted from bone µCT.
- Join Points: This method is used to generate beam-based structures. The first step is common to all variants and consists of generating random points, also called nodes, in a desired volume. These nodes are then joined according to certain criteria. Generally, the objective of this criterion is to control the nodal connection or the geometry between connections.
- µCT Scanning: µCT scanning is employed to generate porous structures from existing structures. This method involves scanning a porous structure to obtain its 3D CAD model, which can be used for further analysis and fabrication. Various types of structures, such as polyurethane sponges, cancellous bone scaffolds, human femoral heads, aluminium open-cell foams, realistic trabecular structures and randomly packed beds of glass beads, have been scanned and utilized to generate porous structures.
- Topological Optimization: Topological optimization algorithms based on principal stresses are used to obtain stiffness-optimized porous structures. These algorithms modify the structure based on stress distribution to achieve optimal mechanical properties. This is the unique method proposed to generate self-supported structures.

### 4.3. Answer to RQ3: Regarding RQ2, What Are the 3D Printing Methods Used to Generate Non-Regular Structures?

**Figure 8:**
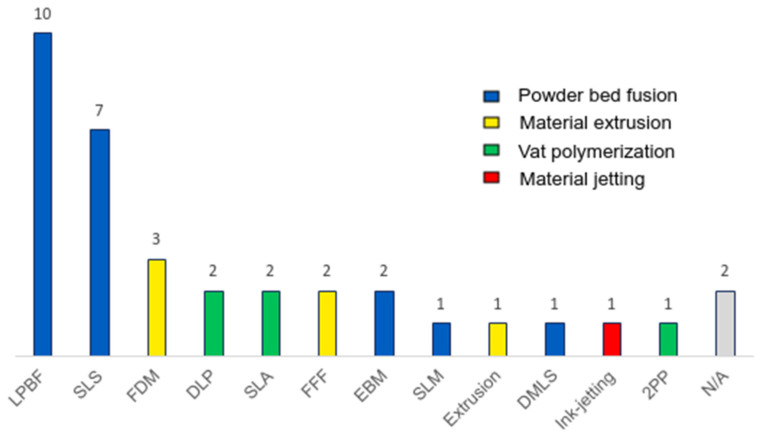
Histogram of AM technologies employed in the analysed articles.

Around 77.8% (n = 35/45) of the stochastic structures generated in the literature are manufactured by 3D printing, while the remaining 22.2% (n = 10/45) of them are just 3D designed. Among all the printed structures, the most used methods are LPBF (10 times), SLS (7 times) and FDM (3 times). Up to 13 printing methods are used to generate stochastic structures, which can be seen in Figure 8, and two articles do not specify which method was used to manufacture the structure [122].

The advantage of metal 3D printing methods over other methods is that they do not require the use of support material during printing. The powder in which the parts are immersed during printing acts as a support. This advantage is ideal for the manufacture of stochastic structures. Due to their complex and angular geometries, the use of support material would be necessary in other methods. Thus, this type of structure constituted the prevailing printing method within the reviewed methodology. However, as shown in Table 8, this method is incompatible with the use of living cells. 

### 4.4. Answer to RQ4. What Proposals Exist for the Generation of Self-Generating Stochastic Structures?

Only 4.7% (n = 6/129) of the articles propose a self-generated structure. Currently, it is clearly an unusual method to generate porous structures because the main trend is to use lattice structures, which are easier to generate. There are no papers that use artificial intelligence (AI) to generate stochastic structures; all the methods used are purely mathematical:

- Topological Optimization: This method is employed by Wu et al. [54] and Martínez et al. [71]. The initial solid structure is subjected to different loads, and topological optimization based on principal stresses is performed. Regions experiencing lower stresses are eliminated to optimize the structure. However, these structures can exhibit anisotropic behaviour due to their dependency on the applied loads. Wu et al. [54] introduce anisotropic filters to mitigate this anisotropy.
- Voronoi Tessellation: Two articles, Alsheghri et al. [130] and Abdullahi et al. [129], utilize Voronoi tessellation to generate stochastic structures. Alsheghri et al. [130] start with an initial structure based on node connectivity data extracted from bone µCT. An iterative optimization process is then conducted by removing the least stressed edges until a convergence criterion is met. In contrast, Abdullahi et al. [129] define a desired volume and pore size and perform Voronoi tessellation to create a stochastic structure that satisfies the design parameters.
- Random Point Generation: The article by Zhang et al. [128] adopts a method where random points are generated within a desired volume. These points are connected following a predefined connection criterion. Coulomb repulsion and Hooke attraction forces are calculated for each node, and node positions are updated until equilibrium is reached, and the optimization process converges.

## 5. Conclusions

There is a great variety of studies on the use of porous structures in different fields, and there is a tendency to use these structures more and more due to the advantages they present such as lightness, reduction of material usage and permeability. For this reason, the applications of these structures are currently diverse and varied, from their use in medicine to the aerospace industry. However, to the best of the authors’ knowledge, no study focuses on the construction of self-supporting bone scaffolds. Therefore, a systematic review was conducted to answer four research questions to capture the current trends in the generation of porous structures. A wide variety of articles proposing different designs of porous structures were analysed. It has been observed that most of them use lattice unit cells to generate such porous structures. Among those using stochastic structures, there was a clear predominance of the use of LPBF techniques to manufacture them. The advantage of these printing methods is that the fabrication of the structures does not require support material, which might be necessary in other methods due to the geometry of the organic structures. Yet neither work meets the authors’ research interests, leaving the door open for future work on the generation of self-supporting bone scaffolds that can be printed by micro-extrusion.

## 6. Future Directions

In this systematic review, various methodologies for designing and 3D printing porous structures have been analysed. Most of them proposed regular lattice structures to generate them, and the most commonly used 3D printing technologies were PBF and material extrusion. As a final objective, this review aimed to search for research that generates self-supporting stochastic porous structures printed with micro-extrusion, or extrusion, that improve the behaviour against crack propagation. Only one paper [65], among all those reviewed, proposed a methodology to create stochastic self-supporting porous structures. However, its methodology focuses on flat structures. Based on the results obtained and the authors’ knowledge, the following future trends are proposed:

- **Design self-supported stochastic patterns:** The results show a lack of research in the development of algorithms capable of generating self-supported stochastic structures that can serve as bone scaffolds. It is true, however, that these scaffolds could be useful in fields other than bioprinting in applications such as thermal dissipation or vibration absorption.
- **Development of AM techniques for bioprinting:** Although many studies analysed generate porous structures that serve as scaffolds, many use printing methods that are not currently compatible with living cells (Table 8). However, under certain conditions, they can be biocompatible. For example, the development of photoinitiators that are not aggressive to cells or use less energetic wavelengths could make many vat polymerisation methods compatible with bioprinting.
- **Hybrid manufacturing techniques:** While there are printing methodologies that could be modified to be biocompatible, there are others, such as PBF, that by their nature could not. But that should not exclude these methods from the field of bioprinting as they could be combined with other methods to promote biocompatibility. It may be interesting to explore the possibilities offered by hybrid printing, where one method prints the scaffold, which provides mechanical strength to the structure, and another method prints the bio-inks, where the living cells could be embedded. This would solve the lack of mechanical strength that biocompatible materials have.
- **Artificial intelligence for designing porous structures:** The use of artificial intelligence in the articles has been considered during the data extraction process. No work has been found that uses AI to generate porous structures. Exploring the capabilities of generative AI to generate this type of structures could be a new methodological approach to obtain, in a simpler way, organic structures more like those present in nature.

## Figures and Tables

**Table 1:** Characteristics of bone tissue cells [10].

Cell	Location	Size	Function
OSTEOCYTES	Cortical bone	7–15 µm	Coordinates the bone remodelling process at all levels
OSTEOBLAST	Periosteum, Endosteum	20–30 µm	Bone tissue formation and maintenance
OSTEOCLAST	Trabecular surface, cortical endosteum	100 µm	Bone resorption
LIMITING CELLS	Endosteum surface	1–2 µm	Activates bone remodelling

**Table 2:** Initial keyword-based searches and results. The searches were performed among the Title, Abstract and Keywords of the articles, combining them with the operator AND.

Keywords (“AND”)	Results
AM, bone	5713
AM, bone, bioprinting	31
AM, bone, bioprinting, auto generative	0
AM, bone, bioprinting, organic structure	0
AM, bone, bioprinting, non-lattice	0
AM, bone, bioprinting, microextrusion	1
AM, bone, bioprinting, 3D	29
AM, bone, bioprinting, generative design	0
AM, bioprinting	150
AM, bioprinting, microextrusion	3
AM, bioprinting, microextrusion, bone	1
AM, organic structures	10
Non-lattice, bioprinting	0
Non-lattice, AM	19
Lattice, bioprinting	71

**Table 3:** Keywords extracted from the preliminary searches.

Category	Keywords
Organic structure	Bone-mimicking, foam, open-cell, porous structure, complex structure.
3D printing	Additive Manufacturing, bioprinting, 3D printing, FDM, geometric modelling.
Non-regular	Bone-like, no-lattice, non-regular, modelling approach, non-parametric design, network-based, graph-based, inhomogeneous structure, irregular internal morphology.

**Table 4:** Inclusion (IC) and exclusion criteria (EC).

Inclusion	Exclusion
IC1: Scientific articles. IC2: 3D porous structures are designed.	EC1: Abstracts, reviews or conference proceedings. EC2: Porous structures are not designed. EC3: Porous structures are not generated by the authors.

**Table 5:** Fields extracted from each article and the research questions they respond to.

Category	Field	Research Question	Extraction Rate
Reference information	- Title - Year - DOI - Authors - Publication source	-	-
Manufacturing	- Is it printed? - Printing method - Material used - Support needed? - Bone related?	RQ3 RQ3 RQ3 RQ2 -	100% 98.4% 97.7% 100% 100%
Infill design	- Lattice regularis - Unit cell type - Pore control	RQ1 RQ1 RQ2	100% 100% 100%
Procedure	- Algorithm used - Software used - AI used - Self-generated - Open source - Code available	RQ2 and RQ4 RQ2 and RQ4 RQ2 and RQ4 RQ4 RQ4 RQ4	100% 62% 100% 100% 100% 100%

The extraction rate is 100% in almost all the fields except for the field *Software used*. This could represent a case of bias for the RQ2 and RQ4. However, this field does not directly address the topic of both questions. Thus, the possible committed bias is reduced.

**Table 6:** Classification of the articles according to the 3D printing technology used.

Family	Method	Articles
Vat polymerization (20)	Twp-Photon Polymerization (2PP) (1)	[16]
	Digital Light Processing (DLP) (7)	[17,18,19,20,21,22,23]
	Direct Laser Writing (DLW) (1)	[24]
	Initiator Integrated 3D printing (i3DP) (1)	[25]
	Stereolithography (SLA) (9)	[26,27,28,29,30,31,32,33,34]
	Projection micro stereolithography (PµSC) (1)	[35]
Material jetting (4)	Binder jetting (2)	[36,37]
	Ink-jetting (2)	[38,39]
Material extrusion (37)	Fused Deposition Modelling (FDM) (16)	[40,41,42,43,44,45,46,47,48,49,50,51,52,53,54,55]
	Fused Filament Fabrication (FFF) (10)	[56,57,58,59,60,61,62,63,64,65]
	Extrusion (6)	[66,67,68,69,70,71]
	µExtrusion (4)	[72,73,74,75]
	Melt Electrowriting (MEW) (1)	[76]
Powder bed fusion (44)	Direct Metal Laser Sintering (DMLS) (2)	[77,78]
	Laser Powder Bed Fusion (LPBF) (16)	[79,80,81,82,83,84,85,86,87,88,89,90,91,92,93,94]
	Selective Laser Sintering (SLS) (12)	[95,96,97,98,99,100,101,102,103,104,105,106]
	Selective Laser Melting (SLM) (8)	[107,108,109,110,111,112,113,114]
	Electron Beam Melting (EBM) (5)	[115,116,117,118,119]
	Multi Jet Fusion (MJF) (1)	[120]
Not specified (2)		[121,122]

**Table 7:** Advantages and disadvantages of different 3D printing methods for the fabrication of porous structures.

Method	Description	Advantages	Disadvantages
Vat polymerization	Uses a UV light source to cure and solidify a photopolymer resin layer by layer.	Ideal for structures with fine features. High detail and smooth surfaces.	Restricted to photopolymer resins. Intricate post-processing.
Material jetting	Deposits droplets of photopolymer material layer by layer, which are then cured using UV light.	Material versatility. Efficient for producing complex structures relatively quickly.	Parts may require additional post-processing. Lower resolution and rougher finish.
Material extrusion	Involves pushing material through a heated nozzle to form layers	Low-cost production. Ease of use.	May require additional support structures. Rougher surface finish.
Powder bed fusion	Uses a laser or electron beam to fuse powdered material layer by layer.	Exceptional mechanical properties. No additional support structures needed.	Significant post-processing required. Very high cost.

**Table 8:** The most relevant aspects of biocompatibility of the main 3D-printing methods.

Method	Biocompatibility
Vat polymerization	Potentially harmful to living cells when using UV light and cytotoxic photoinitiators for curing and solidifying processes.
Material jetting	The conditions that the living cells are exposed to during the ejection can be harmful and incompatible with living cells.
Material extrusion	Suitable for printing living cells as it operates at room or body temperature. Can use hydrogels and bio-inks that provide a supportive environment for cell growth and differentiation. High-viscosity materials require high pressures to extrude, which can be harmful for the living cells.
Powder bed fusion	The high temperatures used in fusing powders are incompatible with living cells. The powder used is not compatible with cell culture.
